# The efficacy of Siddha Medicine, *Kabasura Kudineer (KSK)* compared to Vitamin C & Zinc (CZ) supplementation in the management of asymptomatic COVID-19 cases: A structured summary of a study protocol for a randomised controlled trial

**DOI:** 10.1186/s13063-020-04823-z

**Published:** 2020-10-27

**Authors:** S. Natarajan, C. Anbarasi, P. Sathiyarajeswaran, P. Manickam, S. Geetha, R. Kathiravan, P. Prathiba, M. Pitchiahkumar, P. Parthiban, K. Kanakavalli, P. Balaji

**Affiliations:** 1 Siddha Central Research Institute, Chennai, India; 2 ICMR-National Institute of Epidemiology, Chennai, India; 3 Government Stanley Medical College, Chennai, India; 4 State Licensing Authority (Indian Medicine), Chennai, India; 5 Department of Indian Medicine and Homeopathy, Government of Tamil Nadu, Chennai, India; 6 Central Council for Research in Siddha, Ministry of AYUSH, Chennai, India

**Keywords:** COVID-19, Randomised controlled trial, protocol, Siddha Medicine, Herbal decoction

## Abstract

**Objectives:**

The primary objectives of this study are to determine efficacy of Siddha medicine, *Kabasura kudineer* in reduction of SARS-CoV-2 viral load and reducing the onset of symptoms in asymptomatic COVID-19 when compared to Vitamin C and Zinc (CZ) supplementation. In addition, the trial will examine the changes in the immunological markers of the Siddha medicine against control.

The secondary objectives of the trial are to evaluate the safety of the Siddha medicine and to document clinical profile of asymptomatic COVID-19 as per principles of Siddha system of Medicine.

**Trial design:**

A single centre, open-label, parallel group (1:1 allocation ratio), exploratory randomized controlled trial.

**Participants:**

Cases admitted at non-hospital settings designated as COVID Care Centre and managed by the State Government Stanley Medical College, Chennai, Tamil Nadu, India will be recruited. Eligible participants will be those tested positive for COVID-19 by Reverse Transcriptase Polymerase Chain reaction (RT-PCR) aged 18 to 55 years without any symptoms and co-morbidities like diabetes mellitus, hypertension and bronchial asthma. Those pregnant or lactating, with severe respiratory disease, already participating in COVID trials and with severe illness like malignancy will be excluded.

**Intervention and comparator:**

Adopting traditional methods, decoction of *Kabasura kudineer* will be prepared by boiling 5g of KSK powder in 240 ml water and reduced to one-fourth (60ml) and filtered. The KSK group will receive a dose of 60ml decoction, orally in the morning and evening after food for 14 days. The control group will receive Vitamin C (60000 IU) and Zinc tablets (100mg) orally in the morning and evening respectively for 14 days.

**Main outcomes:**

The primary outcomes are the reduction in the SARS-CoV-2 load [as measured by cyclic threshold (CT) value of RT-PCR] from the baseline to that of seventh day of the treatment, prevention of progression of asymptomatic to symptomatic state (clinical symptoms like fever, cough and breathlessness) and changes in the immunity markers [Interleukins (IL) 6, IL10, IL2, Interferon gamma (IFNγ) and Tumor Necrosis Factor (TNF) alpha]. Clinical assessment of COVID-19 as per standard Siddha system of medicine principles and the occurrence of adverse effects will be documented as secondary outcomes.

**Randomisation:**

The assignment to the study or control group will be allocated in equal numbers through randomization using random number generation in Microsoft Excel by a statistician who is not involved in the trial. The allocation scheme will be made by an independent statistician using a sealed envelope. The participants will be allocated immediately after the eligibility assessment and informed consent procedures.

**Blinding (masking):**

This study is unblinded. The investigators will be blinded to data analysis, which will be carried out by a statistician who is not involved in the trial.

**Numbers to be randomised (sample size):**

Sample size could not be calculated, as there is no prior trial on KSK. This trial will be a pilot trial. Hence, we intend to recruit 60 participants in total using a 1:1 allocation ratio, with 30 participants randomised into each arm.

**Trial status:**

Protocol version 2.0 dated 16^th^ May 2020. Recruitment is completed. The trial started recruitment on the 25^th^ May 2020. We anticipate study including data analysis will finish on November 2020. We also stated that protocol was submitted before the end of data collection

**Trial registration:**

The study protocol was registered with clinical trial registry of India (CTRI) with CTRI/2020/05/025215 on 16 May 2020.

**Full protocol:**

The full protocol is attached as an additional file, accessible from the Trials website (Additional file 1). In the interest in expediting dissemination of this material, the familiar formatting has been eliminated; this Letter serves as a summary of the key elements of the full protocol. The study protocol has been reported in accordance with the Standard Protocol Items: Recommendations for Clinical Interventional Trials (SPIRIT) guidelines (Additional file 2).

**Supplementary information:**

**Supplementary information** accompanies this paper at 10.1186/s13063-020-04823-z.

## Supplementary information

**Additional file 1.** Full Study Protocol.

**Additional file 2.** SPIRIT 2013 Checklist: Recommended items to address in a clinical trial protocol and related documents.

## Data Availability

All patient data will be kept confidential and personal identifiers of the study participants will not be disclosed to the public. Only the study investigators will have access to the study data.

