# Supplementary material for: The efficacy of Siddha Medicine, Kabasura Kudineer (KSK) compared to Vitamin C & Zinc (CZ) supplementation in the management of asymptomatic COVID-19 cases: A structured summary of a study protocol for a randomised controlled trial
Source: Trials. 2020 Oct 27;21:892. doi: 10.1186/s13063-020-04823-z (PMC7590253; doi:10.1186/s13063-020-04823-z)
Supplement: Supplementary file 1 — Additional file 1. Full Study Protocol. [file 13063_2020_4823_MOESM1_ESM.pdf]

A prospective, single centre, randomized open labelled comparative clinical study to evaluate the effectiveness of Siddha medicine, *Kabasura kudineer* and vitamin c-zinc supplementation in the management of asymptomatic COVID 19 patients.{1}

Study protocol registered at Clinical Trial Registry of India with CTRI/2020/05/025215 {2a and 2b}

Protocol Version 2.0 dated 14-05-2020 {3}

Dr. S. Natarajan, M.D(Siddha**).,**
Head, Department of Clinical Research,
Siddha Central Research Institute (SCRI) (CCRS)
drnatarajan78@gmail.com

Dr. C. Anbarasi., M.D(Siddha),
Research Officer (Siddha), Siddha Central Research Institute (SCRI) (CCRS)
dranbu1208@gmail.com

Dr. P. Manickam BSMS., PhD
Scientist E, National Institute of Epidemiology

Dr. P. Sathiyarajeswaran., M.D(Siddha).,
Assistant Director in charge, Siddha Central Research Institute

Dr. Geetha., MD(Gen Med)
Professor, Department of Medicine, Government Stanley Medical College

Dr. Kathiravan., MD(Gen Med)
Assistant Professor, Department of Medicine, Government Stanley Medical College

Dr. Pratheepa., MD(Gen Med)
Assistant Professor, Department of Medicine, Government Stanley Medical College

Dr. P. Parthiban., M.D(Siddha)
Joint Director
Directorate of Indian Medicine and Homeopathy, Government of Tamil Nadu

Dr. K. Kanakavalli., M.D(Siddha)
Director General, Central Council for Research in Siddha, Ministry of AYUSH

Dr. P. Balaji., MS, FRCS, PhD, FCLS
Dean, Government Stanley Medical College

Funding Body/ Sponsor {4}: Central Council for Research in Siddha

Roles & Responsibilities of the investigators {5a}:

Investigators	Roles & Responsibilities
**Natarajan<br>Anbarasi<br>Manickam**	**Study and concept development**
**Natarajan<br>Anbarasi<br>Manickam<br>Sathiyarajeswaran<br>Balaji**	**Methodology and Study design**
**Natarajan<br>Anbarasi<br>Geetha<br>Kathiravan<br>Pratheepa**	**Study conduction**
**Parthiban<br>Kanakavalli<br>Balaji**	**Study supervision**
**All**	**Refinement of study protocol**

Trial sponsor {5b): Central Council for Research in Siddha

Role of study sponsor and funders {5c}:
The funds from the granting will be used in procuring the COVID-19 kits, CBA biomarker kits and laboratory consumables etc. Funders will have no role in study design; data collection, management, analysis, and interpretation of data and writing or dissemination of the final report.

Trial Coordination Center {5d}: Siddha Central Research Institute, Chennai

A prospective, single centre, randomized open labelled comparative clinical study to evaluate the effectiveness of Siddha medicine, *Kabasura kudineer* and vitamin c-zinc supplementation in the management of asymptomatic COVID 19 patients.

Introduction: {6a}

Coronavirus disease 2019 (COVID-19) is a respiratory tract infection caused by a newly emergent coronavirus, SARS-CoV-2, that was first recognized in Wuhan, China, in December 2019. While most people with COVID-19 develop mild or uncomplicated illness, approximately 14% develop severe disease requiring hospitalization and oxygen support and 5% require admission to an intensive care unit.(1) In severe cases, COVID-19 can be complicated by acute respiratory disease syndrome (ARDS), sepsis and septic shock, multiorgan failure, including acute kidney injury and cardiac injury. The mortality rate of COVID-19 is still increasing in other countries than China now. At present, there is limited evidence from randomized clinical trials to support any vaccines or pharmacological treatments from conventional medicine for COVID-19.(2)

According to Siddha system of medicine, the symptoms and signs of COVID-19 are identified as the aggravation of Iyam and which later associated with other Uyir thathukkal Vali and Azhal and expressed as Thontham (Mukkutram) leads to sanni.(3) Siddha medicine has played a major in controlling the mortality rate of chikungunya and dengue in Tamil Nadu by administration of Nilavembu Kudineer during 2015.(4) Siddha medicine has contributed in lowering health burden during public health emergency. Siddha Medicine has a good potential to combat COVID-19(5).

Rationale: {6b}

In this context, one of the classical formulations from Siddha system of medicine is Kabasura kudineer (KSK) consists of 15 herbals ingredients which individually has anti-viral activity (6). Cucurbitacin B (-112.09), Cardiofoliolide (-111.5), Apigenin (-98.84) and Pyrethrin (-92.98) presented in the KSK were found to be effective in preventing novel corona virus binding and replication.(7) Determination of organoleptic characters, preliminary phytochemical analysis, physico- chemical analysis, TLC photo documentation and HPTLC fingerprint studies on KSK were analyzed.(8) Toxicological study on KSK showed that it is safe(9). KSK also possess anti pyretic, anti-inflammatory and anti-bacterial effect. Vitamin c and zinc supplementation(CZ) is being prescribed for the COVID19 positive asymptomatic patients admitted in isolation ward. The role of CZ in the management of COVID19 is still not clear. Therefore, we propose to compare the effect of KSK and CZ in terms of negative conversion of SARS CoV- 2 infection.

Objectives

Primary:{7a}

- o To determine efficacy of KSK compared with CZ in reducing the onset of clinical symptoms in asymptomatic patients
- o To determine effectiveness of KSK compared with CZ in terms of reduction of SARS-CoV-2 viral load from the baseline
- o To examine the effect of KSK compared with CZ on immune system in terms of selected immunological markers

Secondary:{7b}

To document

- Reduction in the risk of disease progression
- Accelerated recovery
- Reduction in intensive supportive care and long-term hospitalization
- Clinical profile in terms of Siddha System of Medicine (SSM)
- Adverse events/effects if any

Proposed methods

Study design {8}:

- Randomized, open-label, clinical trial

Study Setting {9}:

COVID Care Centre managed by Government Stanley Medical College, Chennai

Eligibility criteria {10}
Study participants
Inclusion

1. Laboratory confirmed COVID-19 without symptoms
2. Aged 18-55 years
3. Consenting to participate in the study and sign the informed consent

Exclusion

1. Patient with co morbid conditions like DM, HT, BA
2. Patients with severe primary respiratory disease or other pathogenic microbial pneumonia that needs to be identified with COVID-19
3. Pregnant and mothers, those who have a pregnancy plan.
4. Patients with other systemic malignant diseases such as malignant tumors, mental illnesses, etc., which the researchers consider unsuitable for participation in the study
5. People who have been allergic to Siddha medicine or intolerant to taking medicine
6. Patients participating in other COVID-19 clinical trials

Interventions Arm -1{11a}

Kabasura Kudineer (Siddha Polyherbal decoction)

Table 1: Composition of KSK

S. No	Botanical name	Family	Part Used	Parts
1.	*Zingiber officinale*	Zingiberaceae	Rhizome	1 part
2.	*Piper longum*	Piperaceae	Fruit	1 part
3.	*Syzygium aromaticum*	Myrtaceae	Flower bud	1 part
4.	*Anacyclus pyrethrum*	Asteraceae	Rhizome	1 part
5.	*Tragia involucrata*	Euphorbiaceae	Leaves	1 part
6.	*Solanum anguivi*	Solanaceae	Leaves	1 part
7.	*Terminalia chebula*	Combretaceae	Fruit rind	1 part
8.	*Justicia adathoda*	Acanthaceae	Leaves	1 part
9.	*Anisochilus carnosus*	Lamiaceae	Whole plant	1 part
10.	*Costus speciosus*	Costaceae	Rhizome	1 part
11.	*Tinospora cordifolia*	Menispermaceae	Whole plant	1 part
12.	*Clerodendrum serratum*	Verbanaceae	Leaves	1 part
13.	*Andrographis paniculata*	Acanthaceae	Whole plant	1 part
14.	*Cyperus rotundus*	Cyperaceae	Rhizome	1 part
15.	*Sida acuta*	Malvaceae	Whole plant	1 part

SOP for preparation: To 5g of KSK coarse powder, add 240 ml of water and allowed to boil till, it reduces to one-fourth (60ml) and filter the decoction (KSK).

Dose : 30 – 60 ml twice daily.
Dosage form : *Kudineer* (decoction).
Route of administration : Oral.
Time of administration : twice a day- Morning and Night before food
Duration of therapy : 14 days
Follow up period : 14 days after treatment

Procurement of study drug : KSK will be procured from the GMP certified pharmacy.

Intervention Arm -2

Nutritional Supplement : Vitamin C + Zinc supplementation

Dose : **Zinc -100 mg, Vit C – 60000 IU**
Dosage form : **tablet**
Route of administration : **oral**
Time of administration : **once a day (after food)**
Duration of therapy : 14 days
Follow up period : 14 days after treatment
Procurement of study drug : Government Stanley Medical college

Discontinuation of intervention {11b}: Patients who develop any adverse reactions or patients who do not wish to continue will be discontinued in the study.

Compliance on intervention {11c}: The investigator will dispense the decoction and tablets ensuring the compliance of the drug administration. All the participants will be asked to take medicine in front of the investigator

Concomitant care {11d}: As all the participants are asymptomatic, there is no need for concomitant care

Primary outcomes {12}

- Reduction in incidence of clinical symptoms like fever, cough and breathlessness
- Reduction in Viral load of SARS-CoV-2 at the end of treatment(0,7,14 days)
- Negative conversion of SARS-CoV-2 by 14 days
- Examine the levels immune markers and inflammatory markers (IgG, IgM, IgA, Th1(TNF-Alfa, IFN-gamma, IL2) Th2(IL6, IL10) at the end of treatment(0,7,14 days)

Secondary outcomes

- Reduction in incidence of complications
- Reduction of in hospital time
- Laboratory markers (Laboratory Markers In COVID-19 Patients (10)
- Adverse events/effects

Siddha-based measurements

- Siddha *Udaliyal* assessment
- Siddha *Uyir thathukkal and udal thathukkal assessment*

Table 2: Schedule of Events- Part I {13}

Timepoint	D0	D1	D2	D3	D4	D5	D6	D7	D8	D9	D10	D11	D12	D13	D14
Enrolment															
Eligibility Screen	✓														
Informed Consent	✓														
History of illness	✓														
Intervention		✓	✓	✓	✓	✓	✓	✓	✓	✓	✓	✓	✓	✓	✓
Assessments															
Clinical Symptoms		✓	✓	✓	✓	✓	✓	✓	✓	✓	✓	✓	✓	✓	✓

SSM principles		✓	✓	✓	✓	✓	✓	✓	✓	✓	✓	✓	✓	✓	✓
Laboratory Markers		✓						✓							✓

Sample size {14}
As there is no prior work on this research question, so no assumptions for the sample size calculation could be made. The present study will serve as a pilot trial. We intend to study 60 patients, each 30 in two groups.

Randomization, sequence allocation & allocation concealment{16a,b,c}
Simple randomization. All the eligible participants will be randomly assigned (in 1:1 ratio) in each arm to ensure balanced distribution between KSK and CZ arms. Randomization will be implemented by independent statistician who will not involve in data analysis. Equal numbers of cards with each arm assignment number randomly generated by computer will be placed in sequentially numbered envelops that will be opened on patient enrolling.
Blinding (masking){17a,b}

- No blinding.

Specimen collection details {18a}
Appropriate clinical sample will be collected by laboratory personnel/ health care worker trained in specimen collection in presence of a clinician by following all biosafety precautions and using personal protective equipment (PPEs), clinical samples will be sent to the designated laboratory (ICMR empanelled) by following standard triple packaging.

Table 3: Sample collection details

Specimen type	Collection materials	Transport to Laboratory	Storage till testing
Nasopharyngeal and oropharyngeal swab	Dacron or polyester flocked swabs*	4 °C	≤5 days: 4 °C >5 days: -70 °C
Serum	Serum separator tubes (adults: collect 3-5 ml whole blood)	4 °C	≤5 days: 4 °C >5 days: -70 °C

*For transport of samples for viral detection, VTM (viral transport medium) will be used

Specimen labelling and processing
Personal protective equipment (apron, hand gloves, face shield, N95 Masks etc.) will be used and all biosafety precautions will be followed so as to protect individuals and the environment. Proper labelling (name/age/gender/specimen ID) will be done on specimen container and other details of sender (name/address/phone number) on the outer container by mentioning “KSK_COVID_trial”

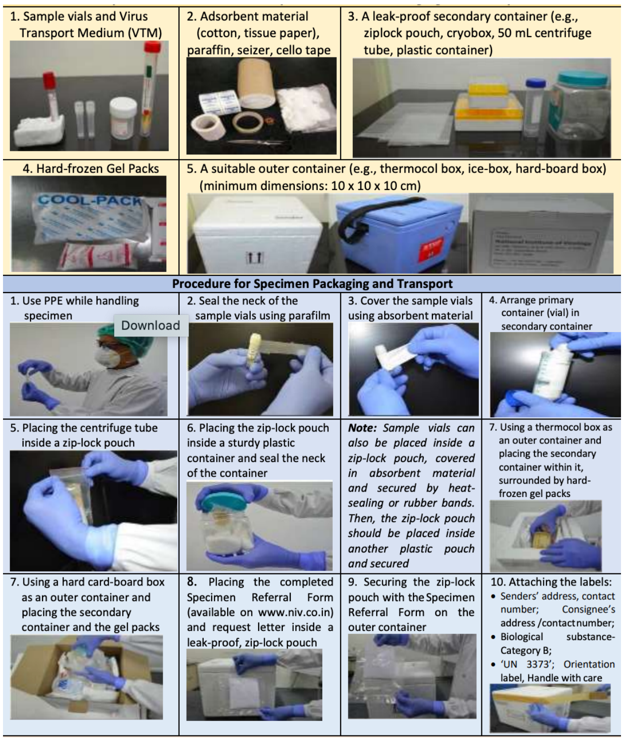

Figure 1: Lab Specimen collection

Laboratory sample collection Schedule: (i) Oropharyngeal swabs (0,7,14 days) - for SARS-CoV-2 RT-PCR (samples will be transported to Laboratory, as per the guidelines); (ii) Blood sample (0,7,14 days) (iii) ECG, Xray (Base line)

All samples would be stored for future-related tests.

Table 4: Schedule of investigations

Parameter	D0	D7	D14
Haemogram$	✓	✓	✓
*LFT	✓		✓
#RFT	✓		✓
HbA1c, BS	✓		
qRT- PCR for SARS Cov2	✓	✓	✓
Electrolytes	✓	✓	✓
Chest X-ray	✓		
ECG	✓		✓
Immunoglobulins	✓	✓	✓

$Hb%, total leucocyte count and differential WBC -neutrophils, lymphocytes, eosinophils, monocytes and basophils, RBC count, platelet count; #Renal function test -BUN, Creatinine; *Liver function test -albumin, bilirubin, ALT, AST, alkaline phosphatase. AST, aspartate transaminase; ALT, alanine aminotransferase; RBC, red blood cell; WBC, white blood cell; BUN, blood urea nitrogen; ECG, electrocardiogram; SARS-CoV-2, severe acute respiratory syndrome coronavirus 2; qRT-PCR, real-time reverse transcription-polymerase chain reaction; HbA1c, haemoglobin A1c; Hb, haemoglobin

Frequency and duration of monitoring: (i) Patients will be monitored daily until discharge from the hospital and followed up till 14 days;

Outcome assessment

Clinical outcomes: (i) Assessment of clinical symptoms (ii) Clinical profile of SSM (iii) Time to test COVID-19 Negative

Safety Assessment: (i) Elevation of ALT to more than five-fold upper normal limit; (ii) Anaphylaxis; and (iii) Adverse events and serious adverse events.

Laboratory outcomes: (i) Viral RNA loads and cycle threshold values in serial samples of nasopharyngeal

and oropharyngeal swabs and blood, collected every week (to document anti-viral activity). (ii) Immunoglobulins values in serial samples of serum collected every week (to document immunomodulatory effect)

Data collection {19}

At the time of recruitment, we will collect socio-demographics, history of illness and clinical examination. At every visit we will do systematic clinical examination, determine adherence to interventions and assessment about any complaints and any adverse events. Both allopathic and Siddha physicians will perform clinical examination and collect relevant details according to their systems.

Baseline laboratory investigations: (i) Haemogram; (ii) Liver function tests (LFTs); (iii) Renal function tests (RFTs); (iv) Haemoglobin A1c and blood sugar, (v) RT-PCR for SARS-CoV-2 (respiratory samples: nasopharyngeal swab, oropharyngeal swab, in addition, sputum, bronchoalveolar lavage (BAL), if available); (vi) electrolytes, (vii) Chest X-ray; (viii) Electrocardiogram (ECG) (ix) Immunoglobulins

Data Analysis plan {20}

The analysis plan on the primary and secondary outcome is described in the below table

Table 5: Statistical analysis plan{20a}

Outcome	Hypothesis	Outcome measure	Methods of analysis
**Primary**			
Viral load of SARS-CoV-2	Reduction of Viral Load after intervention	Viral Load	T test
Immunological markers	Improvement	Th1, Th2 cells	T test
**Secondary**			
Reduction in the risk of disease progression	Reduction of complication	Incidence of complication	Kaplan Meier survival analysis
Reduction in intensive supportive care and long-term hospitalization	Reduction of complication	Incidence of complication	Kaplan Meier survival analysis
Clinical profile in terms of Siddha System of Medicine (SSM)		Questionnaire	Chi-square test
Adverse events/effects		Presence of drug related adverse event (time to event)	Kaplan Meier survival analysis

Data monitoring {21a}: The data safety monitoring committee constituted by Ministry of AYUSH will be responsible for the monitoring the data

Harms/ adverse reaction reporting {22}: The adverse events will be documented for each patient and will be reported to Pharmacovigilance cell of Siddha Central Research Institute, Chennai

Ethical approval {24}: The study will be initiated after the approval of the Institutional Ethics Committee of Government Stanley Medical College, Chennai.

Consent for the trial and biological specimen testing {26}

The purpose of the trial will be explained to all eligible SARS-CoV-2 confirmed patient. Informed consent will be obtained from all eligible participants willing to participate in the trial. Each participant will be informed that participation in the trial is voluntary and that s/he is free to withdraw, without justification, from the trial at any time without consequences and without affecting professional responsibilities. Informed consent will seek approval to collect blood samples and clinical data for the intended purpose of this trial.

Risks and benefits for subjects

This investigation poses minimal risk to participants, involving the collection of a small amount of blood. The direct benefit to the participant is the ability to detect SARS-CoV-2 viral load which would allow for appropriate monitoring and treatment. The primary benefit of the study is indirect in that data collected will help to find an intervention for the management of COVID-19.

Confidentiality{27}
Participant confidentiality will be maintained throughout the investigation. All subjects who participate in the investigation will be assigned a study identification number by the investigation team for the labelling of questionnaires and clinical specimens. The link of this identification number to individuals will be maintained by the investigation team and the CCRS and will not be disclosed elsewhere.

Study will be conducted according to AYUSH GCP guidelines.

Declaration of Interests {28}: All investigators don't have any conflict of interest with the study involved.

References

1. Chan JF-W, Yuan S, Kok K-H, To KK-W, Chu H, Yang J, et al. A familial cluster of pneumonia associated with the 2019 novel coronavirus indicating person-to-person transmission: a study of a family cluster. The Lancet [Internet]. Available from: https://teams.microsoft.com/l/file/F8B7FBC5-C0BC-449D-B8C9-AB76C8E70B10?tenantId= f610c0b7-bd24-4b39-810b- 3dc280afb590&fileType=pdf&objectUrl =https%3A%2F%2Fworldhealthorg. sharepoint.com %2Fsites%2FLibraryCoronavirus2019CoV%2FShared%20Documents%2FGeneral%2FC

2. Chan KW, Wong VT, Tang SCW. COVID-19: An Update on the Epidemiological, Clinical, Preventive and Therapeutic Evidence and Guidelines of Integrative Chinese-Western Medicine for the Management of 2019 Novel Coronavirus Disease. Am J Chin Med. 2020;1–26.

3. Shanmugvelu N. Noi Nadal Noi Mudal Nadal. Chennai: Department of Indian Medicine and Homoeopathy; 1967. 164 p.

4. Anbarasu K, Manisenthil KK, Ramachandran S. Antipyretic, anti-inflammatory and analgesic properties of nilavembu kudineer choornam: a classical preparation used in the treatment of chikungunya fever. Asian Pac J Trop Med. 2011 Oct;4(10):819–23.

5. R. THYAGARAJAN,. Gunapadam (Thathu Seeva Vaguppu). First Edition. Vol. Part II & III. Madras: Directorate of Indian Medicine and Homoeopathy; 1968. 500 p.

6. Vanan T. A Review on " Kapa Sura Kudineer " -A Siddha Formulary Prediction for Swine Flu. 2015 Sep 26;376–83.

7. M P. Coronavirus Spike (S) Glycoprotein (2019-Ncov) Targeted Siddha Medicines Kabasura Kudineer and Thonthasura Kudineer –In silico Evidence for Corona Viral Drug. Asian Journal of Pharmaceutical Research and Health Care,. 2019;Vol 11(2).

8. John A, Jayachandran R, Ethirajulu S, Sathiyarajeswaran P. ANALYSIS OF KABASURAKUDINEER CHOORANAM-A SIDDHA FORMULA-. 2015;3(9):6.

9. Neethu D. Anti-Inflammatory, Antipyretic and Antibacterial Study of Kabasura Kudineer Choornam. In 2017.

10. Lippi G, Plebani M. Laboratory abnormalities in patients with COVID-2019 infection. Clin Chem Lab Med. 2020 Mar 3;
